# Washington City

**Published:** 1863-05

**Authors:** 


					﻿OBITUARY.
Died—In Angelica, Allegany County, April 24th, 1863, of erysipelas
Richard Charles, M. D., aged 65 years and 5 months.
Dr. Charles was born in the County of Tyrone, Ireland; he received
his medical education at Dublin, Glasgow and New York; he emigrated
to America in 1821, and commenced the practice of his profession in the
town of Angelica in 1825, where he continued to reside until the time of
his death. As a medical practitioner Dr. Charles occupied a high and
prominent position. His sound sense, his acknowledged skill as a physi-
cian, his uncompromising hostility to all forms of quackery, his sterling
integrity as a man, his genial disposition and gentle manners will long
continue to be held in grateful remembrance.
Washington City, the National Capital, is undoubtedly in the most in-
sanitary condition of any city in the United States. The principal sources
of uncleanliness are thus given by Dr. Henry G. Glark, of the Sanitary
Commission, in a letter to the military authorities, recommending the
adoption of appropriate measures:—
“ 1st. The accumulation of large numbers of men and animals in con-
fined locations. 2d. The accumulations of filth, such as vegetable and
animal offal, consequent on the above. 3d. The entire neglect of cleansing
operations in the yards, lanes, and streets of the city, especially the very
deficient drainage. 4th. The nuisance of a shallow, and neglected, and
filthy canal in the heart of the city, a receptacle of the sewers, and a place
of deposit for dead horses, etc. 5th. The marshy and stagnant water in
many vacant lots, some of them—as in North Capitol street—ne^r large
hospitals, the want of drainage of which has rendered many parts of the
city, as that near the President’s House, malarious spots, producing inter-
mittent and remittent fevers, jaundice, etc. 6th. The accumulation of the
sick in large numbers is a very powerful means, uuless proper sanitary
measures are taken, of intensifying all the ordinary and extraordinary
causes of disease.”
The recomendwtions of Dr. Clark embrace a rigid system of sanitary
polite, which the nation is interested in seeing enforced.—Medical Times.
				

